# Corexit-EC9527A Disrupts Retinol Signaling and Neuronal Differentiation in P19 Embryonal Pluripotent Cells

**DOI:** 10.1371/journal.pone.0163724

**Published:** 2016-09-29

**Authors:** Yanling Chen, David H. Reese

**Affiliations:** Division of Molecular Biology, Office of Applied Research and Safety Assessment, Center for Food Safety and Applied Nutrition, U.S. Food and Drug Administration, Laurel, MD, 20708, United States of America; Western University, CANADA

## Abstract

Corexit-EC9500A and Corexit-EC9527A are two chemical dispersants that have been used to remediate the impact of the 2010 Deepwater Horizon oil spill. Both dispersants are composed primarily of organic solvents and surfactants and act by emulsifying the crude oil to facilitate biodegradation. The potential adverse effect of the Corexit chemicals on mammalian embryonic development remains largely unknown. Retinol (vitamin A) signaling, mediated by *all-trans* retinoic acid (RA), is essential for neural tube formation and the development of many organs in the embryo. The physiological levels of RA in cells and tissues are maintained by the retinol signaling pathway (RSP), which controls the biosynthesis of RA from dietary retinol and the catabolism of RA to polar metabolites for removal. RA is a potent activating ligand for the RAR/RXR nuclear receptors. Through RA and the receptors, the RSP modulates the expression of many developmental genes; interference with the RSP is potentially teratogenic. In this study the mouse P19 embryonal pluripotent cell, which contains a functional RSP, was used to evaluate the effects of the Corexit dispersants on retinol signaling and associated neuronal differentiation. The results showed that Corexit-EC9500A was more cytotoxic than Corexit-EC9527A to P19 cells. At non-cytotoxic doses, Corexit-EC9527A inhibited retinol-induced expression of the *Hoxa1* gene, which encodes a transcription factor for the regulation of body patterning in the embryo. Such inhibition was seen in the retinol- and retinal- induced, but not RA-induced, *Hoxa1* up-regulation, indicating that the Corexit chemicals primarily inhibit RA biosynthesis from retinal. In addition, Corexit-EC9527A suppressed retinol-induced P19 cell differentiation into neuronal cells, indicating potential neurotoxic effect of the chemicals under the tested conditions. The surfactant ingredient, dioctyl sodium sulfosuccinate (DOSS), may be a major contributor to the observed effect of Corexit-EC9527A in the cell.

## Introduction

The chemical dispersants Corexit-EC9500A and Corexit-EC9527A (abbreviated as Corexit-9500 and Corexit-9527 in this study), listed under the EPA’s National Contingency Plan Product Schedule [1] have been authorized for use in oil spill emergencies in the United States [2]. Both dispersants have been used to mitigate the effect of the 2010 Deepwater Horizon (DWH) oil spill in the Gulf of Mexico. Corexit-9527 was initially used and then replaced by Corexit-9500 in late April 2010 due to the shortage of Corexit-9527 and the evidence that Corexit-9500 was less toxic to aquatic organisms [3]. Throughout the spring and summer of 2010, over 1.8 million gallons of Corexit dispersants were deployed, the largest quantity applied in a single oil spill event [3–5]. In addition to the oil spill, the large-scale usage of chemical dispersants generated considerable environmental and economic impact on the affected areas. Furthermore, the amount of chemicals in the gulf raised concerns over the potential health threat to wildlife and the humans who have been exposed to these chemicals [6–8].

Corexit-9500 and Corexit-9527 are chemical blends primarily composed of surfactants and organic solvents including SPAN80, Tween80, Tween85, propylene glycol, hydro-treated light petroleum distillates, and DOSS (Nalco) [3, 4, 9]. Their exact composition is proprietary information held by the manufacturer. The two formulations differ in that Corexit-9527 contains, whereas Corexit-9500 lacks, the solvent 2-butoxyethanol [6, 10, 11]. In the Corexit-mediated dispersion process, the surfactant ingredients break up and emulsify the crude oil, improving the oil’s availability to microbial biodegradation.

Toxicity assessments for chemical dispersants often use lethality (LC_50_) as a reference benchmark [3]. In fact, each EPA-approved dispersant must be provided with LC_50_ data from two standard aquatic test species [6, 12]. However, the consequences of exposure to dispersants may not necessarily be lethal. For example, Corexit-9500 can significantly affect the immune [11], neurological [9], cardiovascular [13], and pulmonary [14, 15] systems in the rodent models, but did not cause death and apparent chronic deficits in the tested animals. Moreover, much of the previous research has focused on oil-dispersant mixtures that were found more toxic to the tested organisms than the crude oil or the dispersant alone [16, 17] and for this reason, the toxicity data for the dispersants are very limited [3]. Therefore, an in vitro model that is capable of evaluating the non-lethal effects of the Corexit chemicals on cellular functions would be valuable for the safety assessment of the dispersants especially in higher organisms.

**Fig 1:**
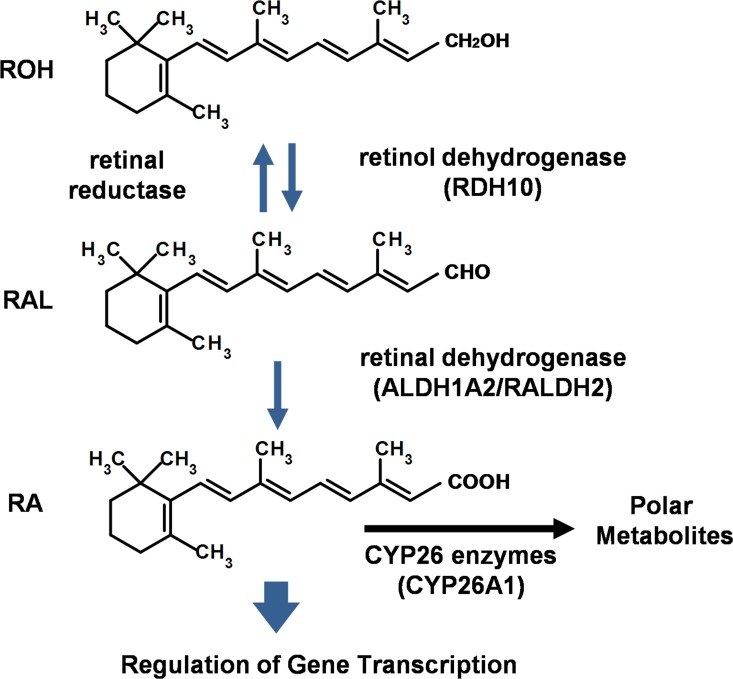
The major regulatory steps in the retinol signaling pathway. The enzymes that are predominantly expressed in P19 cells are listed in parentheses.

RA, the biological active form of retinol/vitamin A (ROH), is essential for embryonic development and many cellular functions in adult animals [18–20]. The physiological homeostasis of RA in cells and tissues is maintained by the RSP, which controls the biosynthesis and catabolism of RA. In the canonical RSP, retinol obtained from the dietary sources is first oxidized by retinol dehydrogenases to retinal (RAL), which is then oxidized by retinaldehyde dehydrogenases to RA (Fig 1) [19, 21]. RA is further metabolized by the Cyp26 cytochrome P450 enzymes for elimination [22]. RA is a potent activating ligand for the RAR/RXR nuclear receptors [23, 24] that regulate the expression of over 500 protein-coding genes [25], such as the *Hox* family of transcription factors that guides the establishment of body patterning along the anterior-posterior axis in the developing embryo [26, 27]. Normal retinol signaling is indispensable for neural tube formation and hindbrain development, as well as the development of the genitourinary tract, eyes, kidneys, diaphragm, lung, limbs and heart [18, 20]. Disruption of the RSP, therefore, is potentially teratogenic [28]. Although some chemical dispersants have been reported to affect development [10, 29–31], the effect of the Corexit dispersants on retinol signaling still remains largely unknown.

In this study, we assessed Corexit-9500 and Corexit-9527 for the effect on retinol signaling using the mouse P19 embryonic stem cell [32, 33], which contains a functional RSP to metabolize ROH to RA [21]. ROH can rapidly induce the expression of the *Hoxa1* gene [21], therefore, chemical interference with the RSP can be reflected in changes in the *Hoxa1* expression [34]. In addition, the P19 pluripotent cell is capable of differentiating into cell types from all three germ layers including neuronal[35, 36], cardiac and skeletal[37], adipose[38], endodermal[39], and endothelial[40] cells. When cultured in the presence of RA, the cell can differentiate into neuron- and glial-like cells [35, 36], representing a good model for studying chemical effect on neuronal differentiation. Based on the aforementioned properties of the P19 cell, a set of in vitro assays was developed to assess the dispersants for cytotoxicity, effect on retinol-induced gene expression, and neuronal differentiation. The results showed that Corexit-9500 was more cytotoxic than Corexit-9527 to this cell. In addition, Corexit-9527 inhibited ROH-induced *Hoxa1* expression by blocking the conversion of RAL to RA. Moreover, Corexit-9527 suppressed ROH-induced P19 cell differentiation into neuron-like and glial-like cells, indicating potential adverse effect on neuronal development. Furthermore, the surfactant component, DOSS, was identified as the major contributor to the effects of Corexit-9527 on the P19 cell. These results provided scientific reference for risk assessment of the Corexit and other chemical dispersants.

## Materials and Methods

### Chemicals

Retinol, retinaldehyde and retinoic acid were obtained from Sigma-Aldrich (St. Louis, MO) and were prepared as 1 mM stock in DMSO and stored in the vapor phase of nitrogen liquid. Corexit-EC9500A and Corexit-EC9527A (Nalco Energy Services, L.P., Sugar Land, TX) were kindly provided by Dr. Paddy Wiesenfeld at the U.S. FDA [41]. SPAN®80 (CAS#1338-43-8), TWEEN®80 (CAS#9005-65-6), TWEEN®85 (CAS#9005-70-3), Dioctyl sulfosuccinate sodium salt (DOSS, CAS#577-11-7), Di-(propylene glycol) butyl ether (CAS#29911-28-2), 2-Butoxyethanol (CAS#111-76-2), and 1,2-Propanediol (CAS#57-55-6) were purchased from Sigma-Aldrich. Chemical concentration of 1ppm is defined as 0.0001% v/v except for DOSS, which is 0.0001% w/v.

### P19 cell

The mouse P19 pluripotent embryonal carcinoma cell line was purchased from the ATCC (Manassas, VA) and maintained in complete MEMα medium (Invitrogen, Carlsbad, CA) supplemented with 10% fetal bovine serum (ATCC) at 37°C and 5% CO_2_. The retinol concentration in serum was 25 nM determined by the supplier.

### Corexit Exposure

Corexit exposure assay was done following a previously described protocol that was used for identifying RSP disruptors [34]. In the short-term (1+6 hr) exposure assay, P19 cells were seeded at 40,000 cells/well, in 100 μl medium, in two 96-well cell culture plates, one for MTT assay (see below) and the other for the *Hoxa1* gene expression assay. After overnight culture at 37°C and 5% CO_2_, the cells were refed with 100 μl of fresh medium containing the desired final concentrations of Corexit. After 1 hr incubation, the cells received 10 μl of retinol-containing medium (final concentration was 0.3 μM) and were cultured for additional 6 hr. The control wells received DMSO (0.03% v/v) without retinol. To terminate Corexit exposure and prepare cell lysate for cDNA synthesis, the wells were washed once in 1x PBS buffer and then 100 μl iScript RT-qPCR Sample Preparation Reagent (BioRad, Hercules, CA) was added. For long-term (24+6 hr) exposure, the assay was similarly carried out except that cells were seeded at 20,000 cells/well and that Corexit incubation time was 24 hr before retinol treatment.

### Cytotoxicity assay

The cytotoxic effect of the tested chemicals was measured using an MTT cell viability assay (ATCC) following the manufacturer’s instructions. The cells in each well received 10 μl of MTT (3-(4,5-Dimethylthiazolyl-2)-2,5-diphenyltetrazolium bromide) reagent, incubated for 2 hr at 37°C and 5% CO_2_, and then lysed in the provided detergent reagent. Absorption at 570 nm was measured on a SpectraMax M^2e^ microplate reader (Molecular Devices, Sunnyvale, CA).

### cDNA synthesis and qPCR

For reverse transcription, 1μl aliquot of iScript cell lysate was used as template in a 10μl reaction containing the SuperScript III reverse transcriptase (Invitrogen) and oligo(dT)_18._ To quantitate gene expression, 1 μl of cDNA was used as template in a 10 μl real-time qPCR reaction run on a Roche LightCycler 480 II (Roche Diagnostics, Indianapolis, IN). The relative change in gene expression was calculated using the 2^-ΔΔCt^ method and *Gapdh* as reference. The primers (500 nM final concentration) for qPCR were: *Hoxa1*: 5’-ccaaaacagggaaagttgga-3’, 5’-gcgctcgtgtaaggtacttgt-3’; *Gapdh*: 5’-aatacggctacagcaacagg-3’, 5’-gcctctcttgctcagtgtc-3’; *Sho1*: 5’-tgcagcagcctcaacgtca-3’, 5’-acctaagagtccatgatgccac-3’; *Tubb3*: 5’-cccgacaactttatctttggtca-3’, 5’-attctcacactctttccgcacga-3’; *Gfap*: 5’-ccaacctccagatccgaga-3’, 5’-cctgcttcgagtccttaatgacc-3’; *Npy*, 5’-acgatgctaggtaacaagcgaat-3’, 5’-gtacccctcagccagaatgc-3’.

### P19 cell embryoid body formation and neuronal differentiation assays

To form embryoid bodies (EBs), P19 cells were seeded at 20,000 cells/plate in 4 mL complete medium in a 60 mm Ultra-Low Attachment culture dish (Corning, Corning, NY), which allows growth of cell aggregates detached from the plate surface. Three culture conditions were used: 0.1% DMSO (the Control), 0.3 μM retinol in 0.1% DMSO, and 0.3 μM retinol in 0.1% DMSO plus 0.01% (100 ppm) Corexit-9527. After 3 days culture at 37°C and 5% CO_2_, EBs were precipitated, washed once in 1x DPBS, dissociated with 2 mL of trypsin (0.25%, Invitrogen) and neutralized using equal volume of soybean trypsin inhibitor (0.5mg/mL, Invitrogen). The dissociated cells were washed once in NBAM medium (Neurobasal-A Medium adjusted to 300 mOsm using NaCl and then supplemented with 2 mM L-Glutamine and 0.25 x B27 Supplement Minus Vitamin A, all from Invitrogen), seeded at 5 x 10^5^ cells/plate (in 2 mL NBAM) in 35mm poly-D-lysine dish (Beckon Dickinson, Franklin Lakes, NJ) and grown for 4 additional days with a change in the medium after 2 days. On day 4, pictures were taken using an Eclipse TE2000-S microscope (Nikon, Melville, NY) and a SPOT RT/KE camera (SPOT Imaging Solutions, Sterling Heights, MI). To measure gene expression, cells were lysed in RNAzol reagent (Molecular Research Centre; Cincinnati, OH) for the isolation of total RNA, which was processed for cDNA synthesis and qPCR analyses as described above.

## Results

### Cytotoxicity of the dispersants

**Fig 2:**
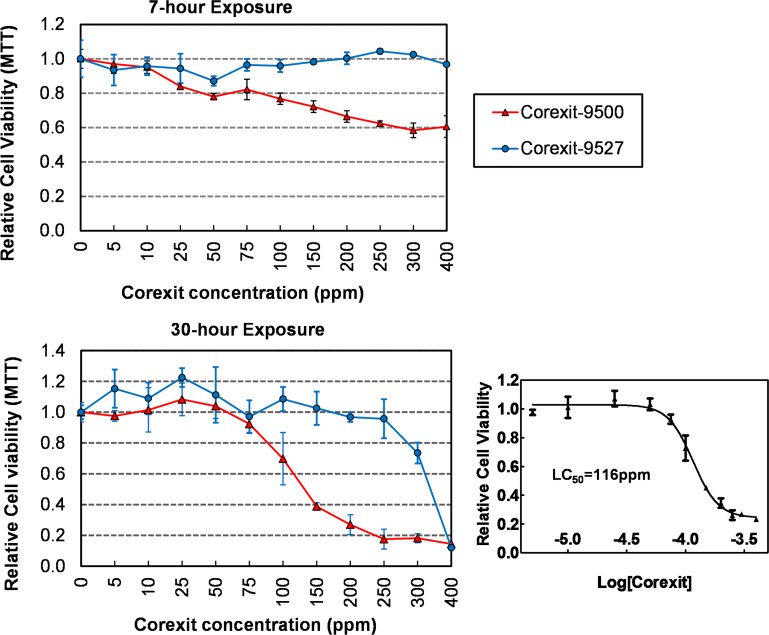
Cytotoxicity of Corexit-9500 and Corexit-9527. P19 cells exposed to dispersants for 7 or 30 hours were measured for viability using the MTT assay. Relative Cell Viability was calculated by normalizing the MTT readings relative to the control cells (no Corexit exposure), which were set to be 1. LC_50_ value for Corexit-9500 was derived from the titration curve using the Graphpad Prism software. Values are mean ± s.e.m.; n = 3.

To assess cytotoxic effects of Corexit-9500 and Corexit-9527, P19 cells were exposed to the dispersants at final concentrations of 0~400 ppm for 7 or 30 hr, which represent the acute or extended exposure times, respectively. In the 7-hr assay, cytotoxicity was observed in cultures that were exposed to Corexit-9500 concentrations of ≥ 100 ppm, whereas Corexit-9527 caused no obvious cytotoxic effect even at the highest tested dose at 400 ppm (Fig 2). In the 30-hr assay, Corexit-9500 significantly reduced cell viability at doses of ≥ 100 ppm with an LC_50_ value of 116 ppm. The Corexit-9527 was cytotoxic at ≥ 300 ppm (LC_50_ value could not be derived from the tested dose range). Results from both the 7-hr and 30-hr assays suggest that Corexit-9500 was more cytotoxic than Corexit-9527 to the P19 cell under the tested conditions, which may result from the different chemical compositions in the two dispersants. Because the primary goal of this research was to evaluate the Corexit dispersants for potential adverse effect on cellular functions without causing cell death, we therefore focused on the less-cytotoxic Corexit-9527 in the following studies.

### Corexit-9527 inhibits ROH-induced Hoxa1 expression

**Fig 3:**
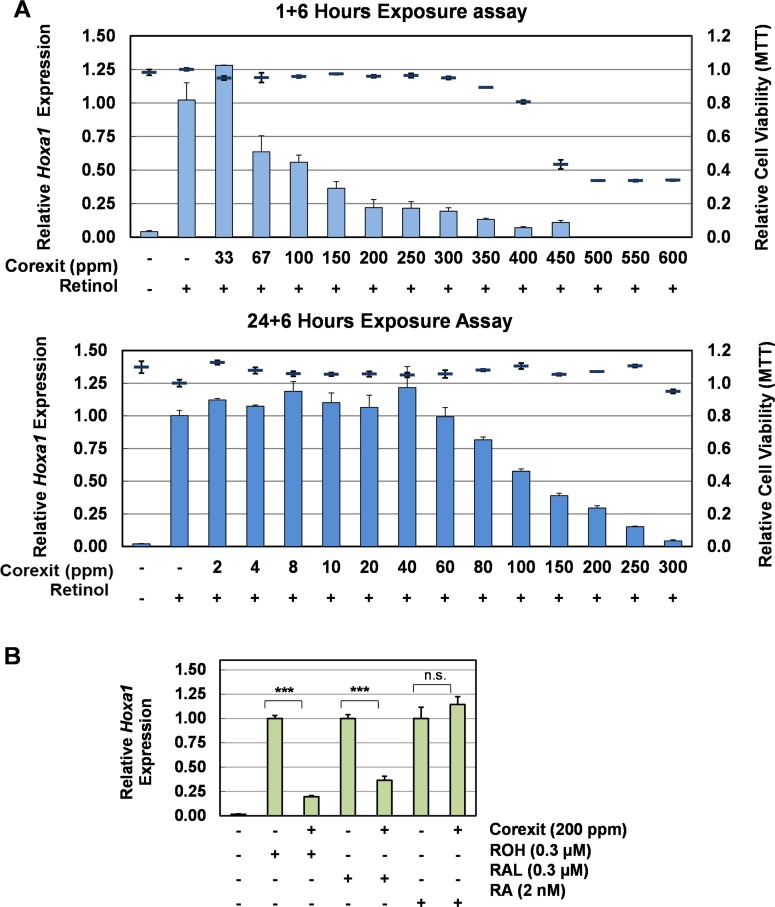
The effect of Corexit-9527 on retinoid-induced *Hoxa1* gene expression in P19 cells. (A) Corexit-9527 inhibited ROH-induced *Hoxa1* expression. P19 cells were exposed to the indicated concentrations of Corexit-9527 for 1 or 24 hr followed by 0.3 μM ROH for additional 6 hr. The columns represent the relative *Hoxa1* expression levels (left axis) that are normalized to the control (no ROH induction). The bars show cell viability (right axis) at the tested doses of Corexit. Values are mean ± s.e.m.; n = 3. (B) The effect of Corexit-9527 on ROH-, RAL- or RA-induced *Hoxa1* expression. P19 cells were exposed to Corexit-9527 for 1 hr and then were induced by indicated retinoids for additional 6 hr. For each retinoid, the *Hoxa1* expression levels in the cells that were induced by the cognate retinoid in the absence of Corexit-9527 were set to be 1. Values are mean ± s.e.m.; ***, *p* < 0.001, n.s., not significant, determined by Student’s *t*-test using two-tailed distribution and unequal variance; n = 4.

The P19 cell contains a functional RSP to metabolize ROH to RA [19, 21]. It also contains the *Hox* gene clusters [26, 27] and the first gene in the *Hoxa* cluster, *Hoxa1*, is a rapid response biomarker of the RSP [34]. To determine the effects of Corexit-9527 on the RSP, we measured ROH-induced *Hoxa1* expression in P19 cells that were exposed to Corexit-9527. In the 1+6 hr exposure assay, Corexit-9527 at non-cytotoxic doses of ≤ 300 ppm significantly inhibited ROH-induced *Hoxa1* expression by a maximum of ~80% (Fig 3A). Similar inhibition was also seen in the 24+6 hr assay where P19 cells were exposed to Corexit-9527 at ≤ 250 ppm. These findings suggest that Corexit-9527 can interfere with retinol signaling and hence RSP-regulated gene expression in the P19 cell.

### Corexit-9527 inhibits the conversion of RAL to RA

In P19 cells, ROH is first oxidized to RAL by the retinol dehydrogenase RDH10 and then to RA by the aldehyde dehydrogenase ALDH1A1/RALDH2 [19, 21] (Fig 1). ROH, RAL and RA all can induce *Hoxa1* expression in P19 cells, albeit their potencies differ: ROH and RAL are of similar potency while RA is approximately 160-fold more potent than ROH [21] (S1 Fig). Therefore, we compared the *Hoxa1* expression induced by the three retinoids at the same efficacy levels in the presence of Corexit-9527 (Fig 3B) to determine whether Corexit-9527 interferes with the oxidation steps in the RSP. At a non-cytotoxic dose of 200 ppm, Corexit-9527 inhibited ROH- and RAL-induced *Hoxa1* expression by ~80% and ~65%, respectively. However, Corexit-9527 did not inhibit *Hoxa1* expression that was induced by 2 nM RA, which has the equivalent induction potency as 0.3 μM ROH and 0.3 μM RAL (S1 Fig). These results suggest that Corexit-9527 primarily interferes with the conversion of RAL to RA in P19 cells and the inhibition on the conversion of ROH to RAL, if exist, is minimal under the tested conditions.

### Effect of Corexit-9527 on P19 cell neuronal differentiation

**Fig 4:**
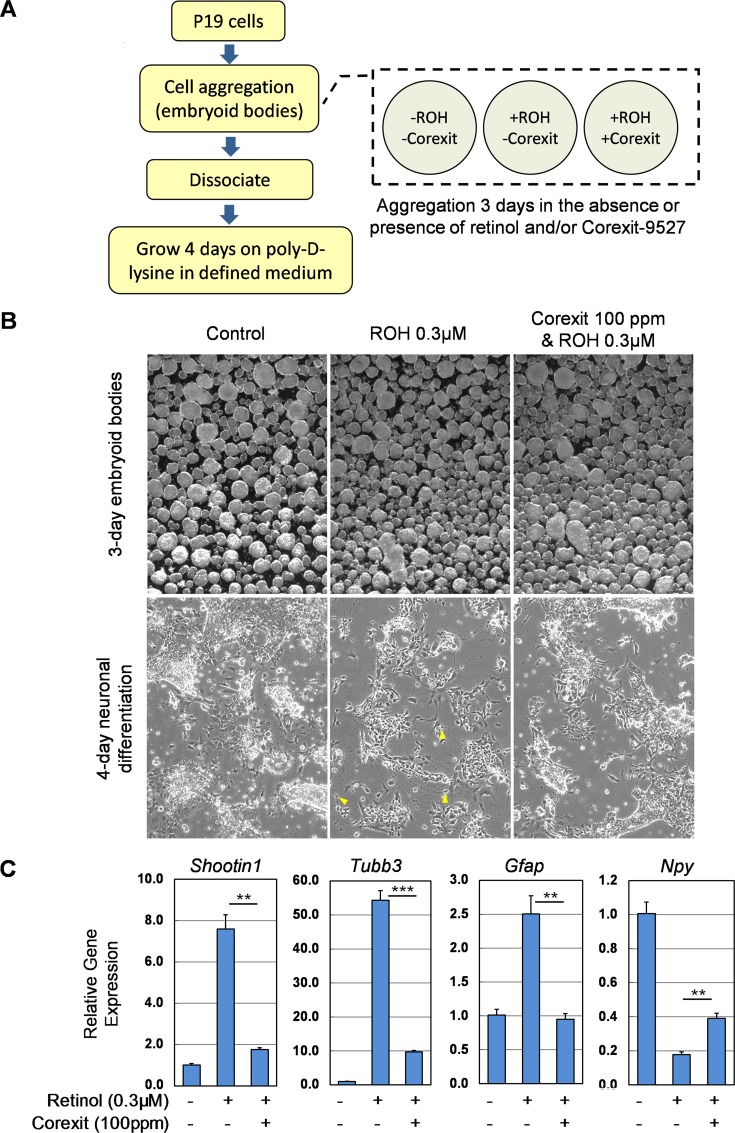
Effect of Corexit-9527 on ROH-induced P19 cell neuronal differentiation. (A) A simplified workflow for the P19 cell embryoid body formation and neuronal differentiation assays. (B) Phenotypical characteristics of the 3-day EBs and the 4-day EBs-derived cells. Yellow arrows indicate examples of neurons. (C) Expression of several neuronal marker genes in the P19-derived cells post EBs formation and differentiation procedures. The control cells were mock-treated with DMSO vehicles only throughout the experimental procedures. Values are mean ± s.e.m.; n = 4. **, *p* < 0.01, ***, *p* < 0.001.

When cultured in suspension as multicellular aggregates (EBs) in the presence of RA, P19 cells can differentiate into neuron- and glial-like cells [35, 36]. Because this cell is capable of metabolizing ROH to RA, ROH can also induce neuronal differentiation as long as the RSP is functional. Conversely, disruption of the RSP by chemicals may attenuate ROH-induced neurogenesis and gliogenesis. To test if Corexit-9527 has an effect on ROH-induced differentiation, P19 cells were exposed to DMSO (vehicle control), ROH, or ROH plus a non-cytotoxic dose of Corexit-9527 (100ppm) for 3 days while EBs were being formed (Fig 4A). The EBs from these three conditions were of similar shape and size after 3 days of culture (Fig 4B) and were subsequently dissociated into individual cells, which were cultured in poly-D-lysine coated plates for additional 4 days. The control cells, which appeared to be morphologically similar to the parental P19 cells, grew rapidly and formed a monolayer with minimal neurite outgrowth (Fig 4B). A large proportion of the cells derived from the ROH-treated EBs appeared to be smaller in size, darker in color, and slower in growth, than the control cells; they showed ample neurite outgrowth that formed an extensive network of neurite fibers (Fig 4B). Interestingly, the cells that were treated with ROH in the presence of Corexit-9527 showed phenotypical characteristics (size, color and growth rate) much similar to those of the control cells. Neurite extension was noticeable but to a much lesser extent compared to the ROH-treated culture (Fig 4B). These results suggest that Corexit-9527, when presented during the time of EBs formation, can interfere with ROH-induced neuronal differentiation in P19 cells.

To further evaluate the consequences of Corexit-9527 interference with ROH-induced P19 cell neuronal differentiation, we examined the expression of several neuronal marker genes including *Shootin1*[42], *Tubb3* (β-tubulin isotype III)[43], *Gfap* (glial fibrillary acidic protein)[44] and *Npy* (Neuropeptide Y)[45, 46]. *Shootin1* has a critical role in synchronized neuronal polarization during development and is required for axon outgrowth [42]. *Tubb3* is a microtubule cytoskeleton component primarily expressed in neurons and is essential for nervous system development and axon maintenance [47]. The expression of the *Shootin1* and *Tubb3* genes was significantly increased in the ROH-treated cells (Fig 4C) but this increase was greatly suppressed in the cells co-exposed to ROH and Corexit-9527. Similarly, the glial cell marker *Gfap* was also up-regulated in ROH-treated cells but Corexit-9527 inhibited such upregulation (Fig 4C). These findings demonstrate that *Shootin1*, *Tubb3* and *Gfap* are ROH-responsive genes, suggesting that they may have important roles in ROH-induced differentiation in P19 cells. NPY is a 36-amino-acid peptide neurotransmitter involved in controlling feeding, obesity, and anxiety/stress-related behaviors [48–51]. *Npy* has previously been shown to be highly expressed in P19 cells [46]. We found that the *Npy* expression was significantly suppressed by ROH treatment and this suppression was partially released by Corexit-9527 (Fig 4C). Consistent with the data from the morphological studies, the results from this gene expression assay demonstrate again that Corexit-9527 has an inhibitory effect on ROH-induced in P19 cell neuronal differentiation.

### Contribution of DOSS to the effect of Corexit-9527 on P19 cells

**Fig 5:**
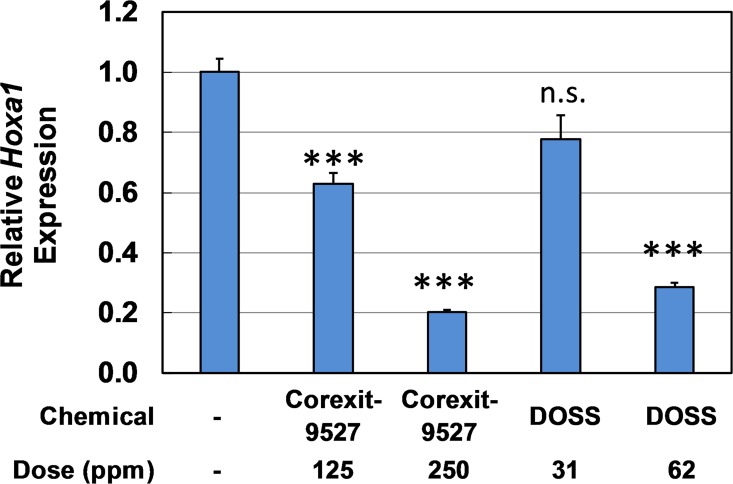
The effect of DOSS on ROH-induced *Hoxa1* expression. P19 cells were exposed to DOSS at 31 or 62 ppm for 24 hr and then induced by 0.33 μM ROH for additional 6 hr. Values are mean ± s.e.m.; ***p < 0.001, n.s., not significant, determined by Student’s *t*-test using two-tailed distribution and unequal variance, compared to the control (left column); n = 4.

Several known components of Corexit-9527 were examined for cytotoxicity in P19 cells during a 30 hr culture period. No cytotoxicity was observed for 1,2-propanediol, 2-butoxyethanol, Span80, TWEEN80, and TWEEN85 at concentrations of up to 500 ppm (S2 Fig and unpublished data). Cytotoxicity was found, however, for the surfactant component DOSS at 125 ppm (and Corexit-9527 at 500 ppm). At a non-toxic dose of 62 ppm (141 μM), DOSS significantly inhibited the ROH-induced *Hoxa1* expression by ~70% (Fig 5), whereas other ingredients did not show such inhibition (data not shown). Although the composition of Corexit-9527 is proprietary, it is known that DOSS accounts for approximately 10~30% of the dispersant by weight (Nalco). Assuming DOSS is the sole active component that is accountable for the observed effect on the P19 cell, the dose of 62 ppm (Fig 5) would be equivalent to 186~630 ppm of Corexit-9527 (corresponding to 30~10% weight of the dispersant) [11, 41]. If 20% (average of 10~30%) of Corexit-9527 is composed of DOSS, then 62 ppm of DOSS should have the same efficacy as 315 ppm Corexit-9527, which is consistent with the results from the cytotoxicity studies (Fig 2). Taken together, these results suggest that DOSS is a major, if not the only, ingredient that is responsible for the observed adverse effects of Corexit-9527 seen in P19 cells. Although other ingredients may not be as effective as DOSS in causing cytotoxicity in P19 cells, their contribution (e.g., additive or synergistic) to interfering the RSP remains to be investigated.

## Discussion

In the present study, the P19 stem cell was used for the first time to evaluate the Corexit dispersants for effect on neuronal differentiation associated with retinol signaling. This pluripotent cell line is self-renewal and can differentiate, under manipulated culture conditions, into cell types from all three germ layers, making it suitable to study the molecular mechanisms that govern embryonic development [32]. Particularly, its ability to differentiate into neuronal cell types upon retinoid treatment [36] supports the application of this in vitro model to study the potential adverse effect of chemicals on neuronal differentiation that is essential for the development of the nerve system. The assays developed in this study can rapidly and cost-effectively assess the chemical dispersants and provide data to gain a better understanding of the mechanism of chemical action.

Corexit dispersants have been shown to affect early developmental stages [10, 29–31, 52–55]. Notably, in March 2011, 151 dead bottlenose dolphins including a relatively high frequency of premature and young animals were found in the northern gulf of Mississippi [56]. The timing of this incident, one year following the DWH oil spill, indicates a potential association between the period of gestational exposure and oil spill, dispersant application, and/or other ecological factors, although such speculation lacks support with direct scientific evidence. Whether Corexit-9527 could influence certain aspects of early development in the dolphins remains speculative and requires further studies.

The Corexit chemicals might act through several possible mechanisms to interfere with retinol signaling and neuronal differentiation in P19 cells. First, the chemicals may directly disrupt the enzymatic function of the ALDH1A2 that converts RAL to RA (Fig 1), which is required for embryo survival and early morphogenesis [57]. Data from this study showed that Corexit-9527 inhibited ROH- and RAL-, but not RA-induced *Hoxa1* expression. Such disruption can lead to reduced *Hoxa1* expression and consequently reduced neuronal differentiation [35]. Second, the Corexit chemicals (e.g., surfactants) may interfere with RAL or ROH binding to the Cellular Retinol Binding Protein 1 (CRBP1) [19], a cytoplasmic protein mainly functions in transporting and delivering retinoids along the enzymatic conversion steps in the RSP [19]. Interference with transporting of retinoids can also reduce RA biosynthesis from the precursors. Third, the signaling across cell membranes that is required for guiding differentiation may be adversely affected by the chemical composition of Corexit. For example, the cell-cell contacts in EBs are primarily established via the Ca^2+^-dependent transmembrane adhesion receptors of cadherins and the surface layer of the spheroid EBs is formed by tight cell-cell junctions [58, 59]. As such, extracellular or endogenous signaling molecules have limited mobility to cross these membranes, forming concentration gradients that are often essential for differentiation to occur [60, 61]. The surfactant ingredients in the dispersants may alter membrane permeability [62] and disturb the gradient diffusion of morphogenic cues, such as RA[61], and consequently inhibit differentiation from proceeding. For example, Corexit-9500 has been reported to increase the permeability of respiratory epithelium in zebrafish gill and the epithelial monolayer in human bronchial airway [63]. The molecular targets of the Corexit chemicals and the exact mechanism remain to be elucidated.

It has been well established that the experimental conditions selected for toxicity assessment for the chemical dispersants, such as exposure duration, temperature, dose and target species, can greatly affect the test results [6, 16, 64, 65]. It is common that chemical dispersants are assessed for toxicity (LC_50_) in 24–96 hr exposure times [3]. However, a 4-hr exposure time period is thought to be more reflective of a true acute exposure scenario [64], since dispersion process lasts for only a short period of time before the chemicals are dissolved in water columns. Therefore, the 7-hr and 30-hr exposure time periods for the cytotoxicity assays and the 72-hr exposure time for the differentiation assays should be adequate to represent the acute and extended exposure scenarios. The assay temperature of 37°C is different from seawater temperatures. However, it is optimal for mammalian cell growth and importantly it reflects the temperature at which mammalian cells are exposed to the chemicals once they enter the body. Finally, it has to be pointed out that the concentrations of the dispersants deployed at sea can be considerably different from those under the laboratory conditions. The EPA and U.S. Geological Survey have closely monitored water and sediment samples following the DWH spill and found no dispersant-related chemical to exceed their aquatic benchmarks, except for one transient case [3, 66]. The National Oceanic and Atmospheric Administration (NOAA) and U.S. FDA laboratories also tested many samples prior to reopening of the Gulf of Mexico for fishing [7, 8]. The concentrations of DOSS, for example, were found to be several orders of magnitude lower [8, 66] than the doses found in this study to inhibit the *Hoxa1* gene expression. Nevertheless, due to the property of the dispersion process, a water column may transiently contain high concentrations of the soluble chemicals especially when the dispersants were applied in large quantities and repeatedly in the same areas [13]. The affected sea animals, therefore, might have been exposed to the chemicals at a relatively high dose or over an extended period of time or both.

In summary, we used in vitro assays based on the P19 stem cell model to assess the Corexit dispersants and found that Corexit-9527 interferes with retinol signaling and neuronal differentiation in this cell. These assays can also be used to study other chemical products [11] for the effects on retinol signaling and neuronal differentiation. Further studies are still needed for understanding the exact mechanism by which the Corexit chemicals, individually or as mixtures, exert influence on embryonic development.

## Supporting Information

S1 Fig P19 cell responsiveness to ROH, RAL and RA induction. **(A)** *Hoxa1* gene expression induced by retinoids for 6 hr. Values were determined by RT-qPCR (see Materials and Methods) and expressed as mean ± s.e.m.; n = 2. The EC_50_ values for ROH, RAL and RA are 100.3 ± 29.7 nM, 117.7 ± 36.1 nM and 0.62 ± 0.16 nM, respectively. **(B)** Relative Luciferase Unit (RLU) expression from reporter plasmids in P19 cells that were induced by retinoids for 6 hrs. ***Experimental procedure*:** P19 cells to be transfected were seeded at 4 x 10^5^/well, in complete medium, in a 6-well cell culture plate and grown overnight at 37°C and 5% CO2. On the 2^nd^ day morning, the cells were transfected with 4 μg pGL3-*RARE-Luc* reporter plasmid (Addgene #13458, Cambridge, MA) with FuGene HD transfection reagent (Promega, Madison, WI) following the manufacturer’s protocols. On the 3^rd^ day (24 hr post transfection), the cells were replenished with fresh medium for recovery for 8 h and then trypsinized and seeded at 4 x 10^4^/well in complete medium in a 96-well cell culture plate. On the 4^th^ day (48 hr post transfection), the cells were induced by retinoids (dose titration) for 6 hr. To quantitate cellular luciferase activity, the cells were lysed using the Luciferase Assay Systems (E1500, Promega) and RLU was measured on a GloMax Multi+ detection system (Promega). The pEGFP-N1 plasmid (Clontech) was used to monitor the transfection efficiency, which was estimated to be >90% under a fluorescent microscope. Information about the pGL3-*RARE*-*Luc* plasmid can be found at Hoffman et al, J Cell Biol. 2006 Jul 3. 174(1):101–13. We thank Michael T. Underhill for sharing this plasmid through Addgene.
(TIF)

S2 Fig Cytotoxicity of several known ingredients of Corexit-9527. P19 cells were exposed to each chemical at indicated doses for 24 hr followed by induction by 0.3 μM ROH for 6 hr (a total of 30 hr). Cell viability was determined using the MTT assay described in the Materials and Methods section. Values are mean ± s.e.m.; n = 4.
(TIF)
