# Supplementary figures and images for: Corexit-EC9527A Disrupts Retinol Signaling and Neuronal Differentiation in P19 Embryonal Pluripotent Cells
Source: PLoS One. 2016 Sep 29;11(9):e0163724. doi: 10.1371/journal.pone.0163724 (PMC5042420; doi:10.1371/journal.pone.0163724)

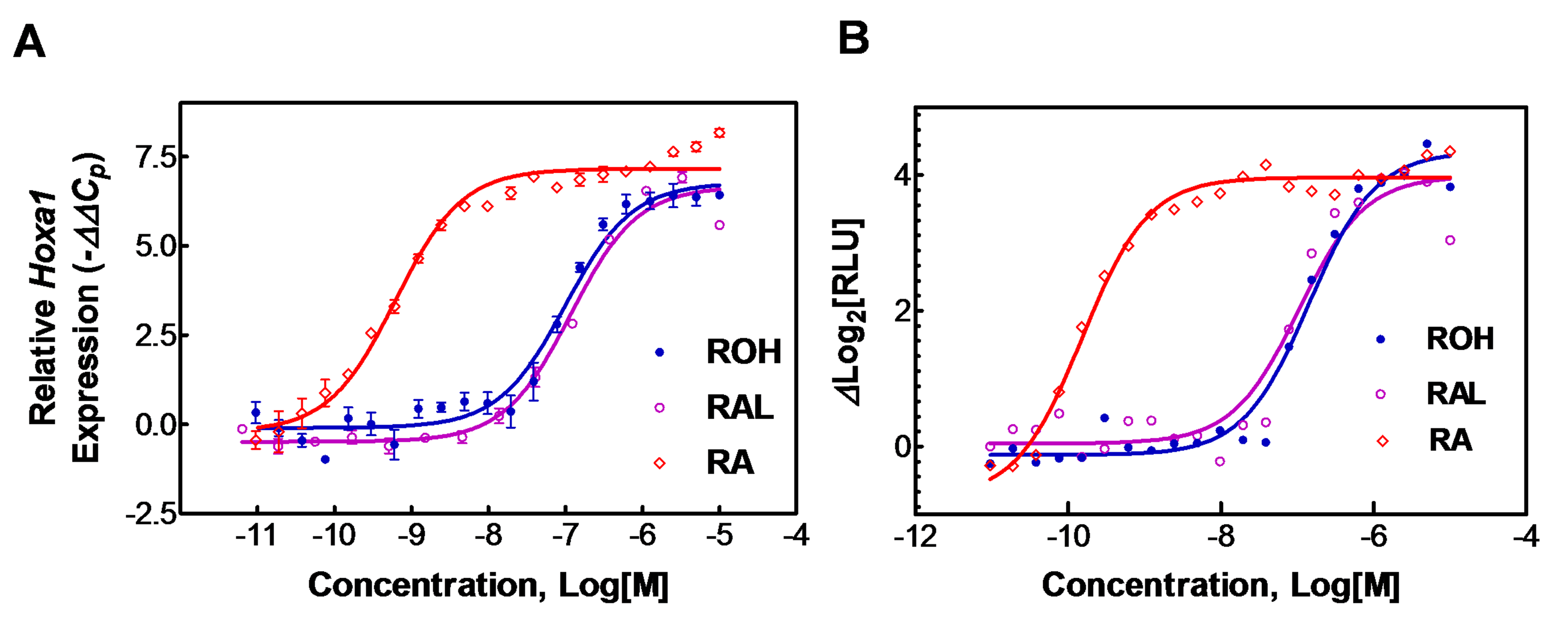

Supplement: S1 Fig — (A) Hoxa1 gene expression induced by retinoids for 6 hr. Values were determined by RT-qPCR (see Materials and Methods) and expressed as mean ± s.e.m.; n = 2. The EC50 values for ROH, RAL and RA are 100.3 ± 29.7 nM, 117.7 ± 36.1 nM and 0.62 ± 0.16 nM, respectively. (B) Relative Luciferase Unit (RLU) expression from reporter plasmids in P19 cells that were induced by retinoids for 6 hrs. Experimental procedure: P19 cells to be transfected were seeded at 4 x 10^5^/well, in complete medium, in a 6-well cell culture plate and grown overnight at 37°C and 5% CO2. On the 2nd day morning, the cells were transfected with 4 μg pGL3-RARE-Luc reporter plasmid (Addgene #13458, Cambridge, MA) with FuGene HD transfection reagent (Promega, Madison, WI) following the manufacturer’s protocols. On the 3rd day (24 hr post transfection), the cells were replenished with fresh medium for recovery for 8 h and then trypsinized and seeded at 4 x 10^4^/well in complete medium in a 96-well cell culture plate. On the 4th day (48 hr post transfection), the cells were induced by retinoids (dose titration) for 6 hr. To quantitate cellular luciferase activity, the cells were lysed using the Luciferase Assay Systems (E1500, Promega) and RLU was measured on a GloMax Multi+ detection system (Promega). The pEGFP-N1 plasmid (Clontech) was used to monitor the transfection efficiency, which was estimated to be >90% under a fluorescent microscope. Information about the pGL3-RARE-Luc plasmid can be found at Hoffman et al, J Cell Biol. 2006 Jul 3. 174(1):101–13. We thank Michael T. Underhill for sharing this plasmid through Addgene. (TIF) [file pone.0163724.s001.tif]

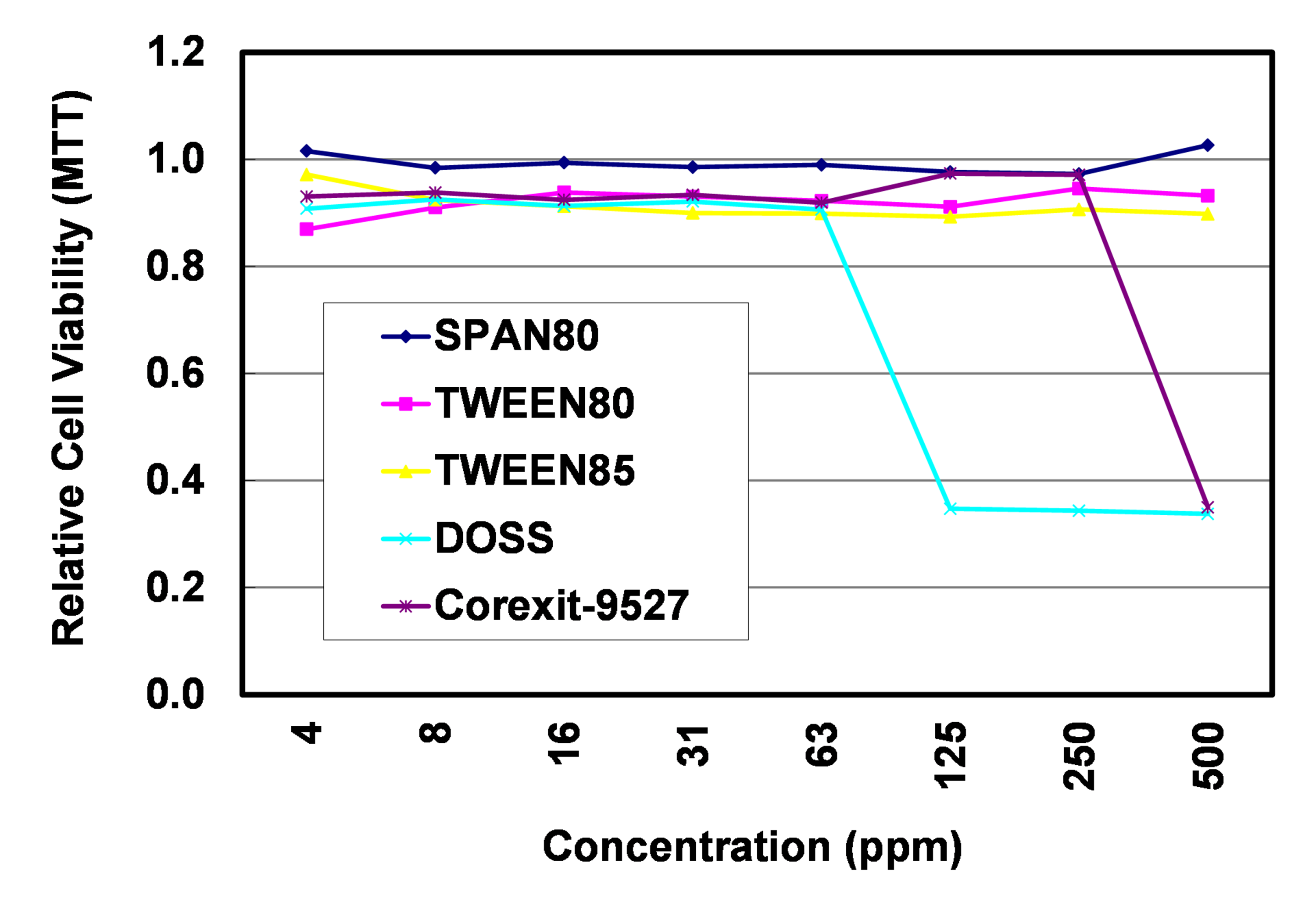

Supplement: S2 Fig — P19 cells were exposed to each chemical at indicated doses for 24 hr followed by induction by 0.3 μM ROH for 6 hr (a total of 30 hr). Cell viability was determined using the MTT assay described in the Materials and Methods section. Values are mean ± s.e.m.; n = 4. (TIF) [file pone.0163724.s002.tif]
